# The differences in learning motivation of college freshmen in Northwest China

**DOI:** 10.3389/fpsyg.2022.997137

**Published:** 2022-10-11

**Authors:** Ke Qiao, Ruizhao Xu, Bailong Liu, Xiangyang Chen, Pan Gu

**Affiliations:** College of Metallurgical Engineering, Xi’an University of Architecture and Technology, Xi’an, China

**Keywords:** college freshmen, learning motivation, personal achievement, material pursuit, gender

## Abstract

The study aimed to investigate the learning motivation of freshmen from a university in Northwest China, which can supply a reference for improving their learning quality and objectives. Data were collected from 800 freshmen of different majors with a learning motivation questionnaire. Differences in learning motivation between different majors, genders, regions, and students are studied. The results show that gender, seeking knowledge orientation, and material pursuit have significant effects on students’ learning motivation. The gender had a significant impact on personal achievement and the only child or not had an obvious effect on material pursuit, while other factors had no obvious difference in gender, regional, and only child or not, while other factors on the gender, regional, and whether the one-child had no obvious difference. According to the results of the research, measures to improve learning motivation are proposed. Our research results provide a reference for improving learning attitude and the quality of universities.

## Introduction

With the rapid development of China’s economy, the demand of social development for high-quality talents is increasing. Meanwhile, the educational resources are gradually enriched, which provides more learning opportunities for college students with more learning opportunities. In 2020, the number of Chinese college students exceeded 8.74 million (Liu et al., 2021). The learning quality of college students has become the focus of social concern, because college students are the main source of talents.

Learning motivation is a driving force for study and is the key factor that determines the quality of learning (Oudeyer et al., 2016; Chang et al., 2020). It is an important factor to motivate students to improve themselves academically and to affect their academic performance (Hu and McGeown, 2020). In order to improve the quality of training, colleges in China have paid more attention to helping students to form healthy learning motivation (Cao and Meng, 2020). Generally speaking, most students have a positive and healthy motivation, but some students have some problems such as addiction to social software or games to drop out, not caring about academic performance, and lack of confidence in learning.

According to Ryan and Deci (2002), motivations can be divided into intrinsic and extrinsic motivations. The intrinsic motivation is the motivation to participate in activities for the reason of the activity itself, but not for some external reward (Chang et al., 2020). The extrinsic motivation is the motivation to participate in activities as the way to achieve a certain goal, such as money, social advancement, friendship or avoid punishment, etc. (Tranquillo and Stecker, 2016). For students, especially freshmen in China, many factors have an effect on their learning motivations due to change in social environment with the development of the economy and technology. This will bring changes in their intrinsic and extrinsic motivations.

Meanwhile, family is the first environment for students (Octaviany and Usman, 2021), and improvement family socioeconomic status will make parents provide more funds for student’s study and opportunities. The only child in a Chinese family will enjoy more opportunities and financial support. Gobena (2018) said that family socio-economic status has an impact on students’ academic performance achievement at college of education and behavioral sciences. At the same time, both external and internal learning motivation will be changed compared with the university students in China’s backward economy, in especial before reform and opening up. Durdy et al.’s (2020) research suggests that the socioeconomic factor does not have a direct effect on learning outcome. However, Kormos and Kiddle’s (2013) research showed that the difference of family and social status has a significant impact on students’ learning motivation.

Besides, education background will also have an impact on students’ learning motivation (Qi and Zheng, 2010). For example, the training objectives of engineering students and art students from primary school to university are inconsistent, and they have differences in personal ideal pursuit, social orientation, and material pursuit. At the same time, gender is also a factor that cannot be ignored. For example, female students are more worried about failure and take learning more seriously (Chang et al., 2020).

In this article, university freshmen with different professional backgrounds were selected to analyze their learning motivations from the effects of family economy, gender, whether they are an only child or not, and professional education background, which will provide a reference for improving learning attitude and the quality of universities.

## Participants and methods

**FIGURE 1:**
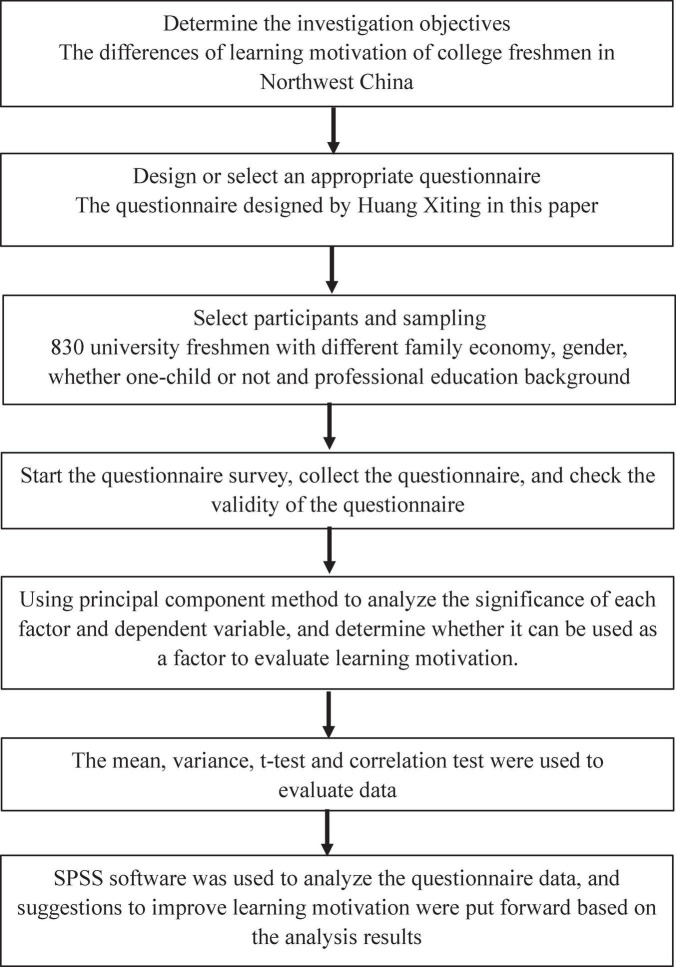
Research flow chart.

The flow chart of this study is shown in Figure 1. The main research objective of this article is to study the differences in learning motivation of college freshmen in Northwest China by questionnaire survey. The participants and the sampling, learning motivation questionnaire, and statistical methods are shown as follows.

### Participants and sampling

The participants were chosen from freshmen studying various majors in different colleges in Shaanxi province in China and had a total of 830. A total of 796 valid questionnaires were collected, with a valid response rate of 95.9%.

### Learning motivation questionnaire

The questionnaire designed by Huang and Zheng (1999) is used to evaluate college students’ learning motivation. According to the survey on the demand structure of Chinese college students by Huang et al., the needs of college students can be divided into six basic types: physiological, safety, communication, respect, development, and contribution. Based on the embodiment of the six needs in learning motivation, 28 kinds of learning motivation are listed and randomly arranged. The college students were required to self-evaluate whether they have such a learning motivation. There are a total of 26 questions about pursuit of knowledge and progress (questions 1–6), social orientation (questions 7–12), material pursuit (questions 13–16), fear of failure (questions 17–20), personal achievement (questions 21–23), and small group orientation (questions 24–26). The choice of each question is the number between 1 to 6, and the number represents the extend of the respondents’ approval of the question involved. The bigger the number is, the higher the approval is.

### Statistical methods

Factors of learning motivation were evaluated with the principal component analysis method in the SPSS version 23.0 software package, and the significance of the factors and dependent variables was studied. KMO metrics were used to determine whether they can be used as factors to evaluate learning motivation. A number of 0.9 or above means very fit, 0.8 means fit, 0.7 means general, 0.6 means not suitable, and anything below 0.5 is an extremely bad fit. The data were evaluated by ways of mean, variance and *t*-test, and correlation test were used to evaluate data. A value of *P* < 0.05 was considered statistically significant.

## Results

### Overall evaluation of learning motivation

The results obtained using the above statistical model are shown in Table 1. It can be seen that the factors in the questionnaire have certain significance in learning motivation. Among them, material pursuit and learning motivation have appropriate significance, while personal achievement, fear of failure and social orientation have general significance with learning motivation, and small group orientation and personal achievement have weak significance with learning motivation.

**TABLE 1:** Mean scores of the six motivations of the respondents and their significance in learning motivation.

The serial number	Factors	Learning goals	On average score	Single problem significance	Factor significance
1	Knowledge and progress	Enrich and enrich their own spiritual world	4.226	0.644	0.700
2		Improve own quality	4.675	0.687	
3		To be successful, one should give full play to one’s potential and value	4.755	0.752	
4		Be interested in the content or the study itself	3.961	0.653	
5		The pursuit of true knowledge, a thorough understanding of the world	4.134	0.679	
6		Future career success	4.838	0.784	
7	Social orientation	Maintain the honor of the school, win glory for the school	3.942	0.702	0.705
8		Class and department honors	3.962	0.696	
9		To be useful and social	4.727	0.709	
10		To serve others while achieving self-worth	4.672	0.745	
11		Response for teacher’s hardworking	4.19	0.718	
12		Human progress and prosperity	4.09	0.657	
13	Material pursuit	Get ahead, get a higher social status	4.621	0.765	0.800
14		Learn to make money	4.737	0.833	
15		To be competent for future work	4.891	0.784	
16		I want to have a rich family after I start a family	4.785	0.818	
17	Fear of failure	Discipline	2.97	0.736	0.725
18		I am afraid I cannot graduate and get a diploma	3.197	0.725	
19		Competition among classmates	3.278	0.711	
20		Afraid of being blamed by friends and relatives for poor grades	3.175	0.727	
21	Personal achievement	Make a career and become famous	4.373	0.662	0.609
22		To gain some kind of power or position in the future	3.706	0.588	
23		Find an ideal life partner	3.991	0.576	
24	Small group orientation	To live up to a man I love very much	4.345	0.766	0.688
25		To repay my benefactor for raising me	4.572	0.707	
26		Argue for a few good friends	3.315	0.59	

### Difference analysis of learning motivation

The control variable method was used to analyze the differences in gender, region, being only child or not, and discipline among the components of learning motivation. The Table 2 shows the significance of learning motivation among students between gender, region, and only child or not. The gender had a significant impact on personal achievement (*P* = 0.001 < 0.05), and the only child or not had an obvious effect on material pursuit (*P* = 0.004 < 0.05), while other factors had no obvious difference in gender, regional, and only child or not (*P* > 0.05). Learning motivation is affected by gender and whether or not a student is an only child.

**TABLE 2:** Significance of learning motivation in gender, region, and only child or not.

Factors	Gender	The average	Gender	Regional	The average	Regional significant	One child or not	The average	Only child or
			significant						not significant
Knowledge and progress	Male	4.409	0.483	Rural	4.213	0.768	Yes	4.357	0.096
	Female	4.468	0.483	City	4.445	0.770	No	4.492	0.101
Social orientation	Male	4.281	0.627	Rural	4.293	0.428	Yes	4.199	0.171
	Female	4.234	0.631	City	4.223	0.436	No	4.318	0.177
Material pursuit	Male	4.779	0.539	Rural	4.819	0.099	Yes	4.619	0.004**
	Female	4.724	0.539	City	4.675	0.104	No	4.873	0.004**
Fear of failure	Male	3.178	0.531	Rural	3.127	0.510	Yes	3.169	0.783
	Female	3.116	0.530	City	3.192	0.510	No	3.142	0.784
Personal achievement	Male	4.139	0.001	Rural	4.068	0.255	Yes	3.957	0.198
	Female	3.833	0.001	City	3.961	0.259	No	4.077	0.202
Small group orientation	Male	4.033	0.061	Rural	4.005	0.398	Yes	3.915	0.115
	Female	3.891	0.061	City	3.943	0.405	No	4.031	0.120

**Significant difference (P < 0.05), only child or not.

### Correlation analysis of the influence of various factors of learning motivation on academic performance

**FIGURE 2:**
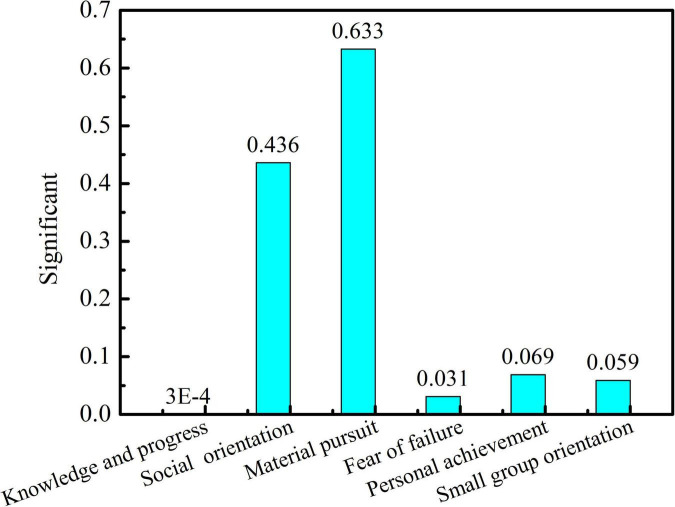
Significance of students’ learning motivation in college of metallurgical engineering, college of mechanical and electrical engineering, and architecture college of Xi’an.

**FIGURE 3:**
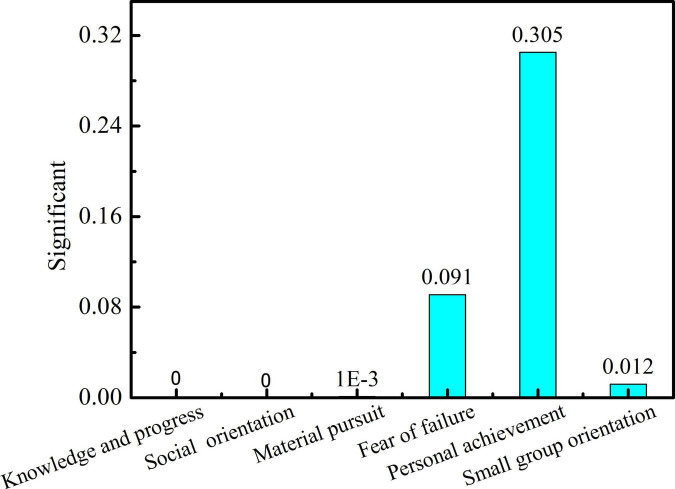
Significance of students’ learning motivation in college of metallurgical engineering, college of mechanical and electrical engineering, and college of art. Table 2 significance of learning motivation in gender, region, and only child or not.

As can be seen in Figure 2, the students of different majors such as metallurgy, mechanical and electrical engineering, and architecture have significant differences in pursuit of knowledge and progress (*P* = 0.0003 < 0.05) and fear of failure (*P* = 0.0031 < 0.05), while there are no significant changes in social orientation, material pursuit, personal achievement, and small group orientation. As can be seen in Figure 3, students from the college of metallurgy and mechanical and electrical engineering and those from the college of arts showed significant differences in intellectual advancement (*P* = 0 < 0.05), social orientation (*P* = 0 < 0.05), and small-group orientation (*P* = 0.001 < 0.05) but no obvious differences in fear of failure and personal achievement.

## Discussion

### Overall evaluation of college students’ learning motivation

From the data analysis in Table 1, it can be seen that the correlation coefficients of various influencing factors of learning motivation are material pursuit, fear of failure, social orientation, knowledge pursuit and progress, small group orientation, and personal achievement from the top to the bottom.

Compared with the ranking of learning motivation of ordinary students, the ranking of material pursuit is basically consistent with the survey results (Xu, 2015). It is worth noting that the results of fear of failure in freshmen’s learning motivation were increased from the sixth place to the second place compared with the previous survey results (Xu, 2015), while the ranking of the other influencing factors of learning motivation did not change that much (Liu et al., 2005; Cheng, 2019). The above results show that the college students generally believe that the main purpose of going to college is to be able to satisfy their material life in the future, and that “to find a good job in the future” and “to have a rich family” are the expectations of the students.

However, fear of failure is rising because at the beginning of the semester, the students have high enthusiasm and strong sense of competition, and strictly abide by the school system, but such a motivation will gradually weaken later, and the fear of failure has no sustainable effect on learning motivation (Du, 2008). Furthermore, instead of being completely immersed in the passive learning of material pursuit and fear of failure, freshmen can show their determination to be a useful person and realize their own value. Compared with the personal achievement, social orientation has a greater impact on students’ learning motivation. This means that students are willing to work hard to maintain the honor of the school, college and class, and to become a person beneficial to society. However, we should also be aware that current college freshmen show insufficient motivation to pursue knowledge and progress, which is manifested by the lack of interest in improving their quality, learning content, and motivation to pursue true knowledge, etc., and that they still look forward to having a successful career.

### Analysis on the difference in freshmen’s learning motivation

As can be seen in Table 2, the learning motivation of the freshmen showed significant differences in gender and whether they are an only child or not, but the differences were not significant in region. Specifically, the male and female students show significant differences in personal achievements, and their motivations for becoming famous and seeking power, status, and ideal lifelong partners are obviously different. However, the male students have a significantly higher desire for personal achievements than the female students. This result is similar with previous research, which pointed out that female students have learning motivations different from those of male students (Chang et al., 2020).

Although the learning motivation of college freshmen is not obviously different in regions, and the learning motivation of rural students in social orientation, material pursuit, personal achievement, and small group orientation is higher than that of urban students. Maybe it is because students in rural areas are at a resource disadvantage. After entering universities, rural students show a strong desire to learn and strive for honor or material security for themselves and the collective through their own efforts (Oudeyer et al., 2016; Qi and Zheng, 2010). Urban students, on the other hand, prefer to improve their personal quality and achieve career success.

In Figures 2, 3, it can be seen that significant differences between engineering students (metallurgy and mechanical and electrical) and art-related subjects (architecture and art) are reflected in the orientation of seeking knowledge. This may be due to differences in disciplines and students’ training modes. The engineering students are good at theoretical subjects but lack thinking skills (Kuo et al., 2021). However, the students majoring in arts need to study independently or cooperatively, and they need to pursue their own potential in learning, thus stimulating creativity. Moreover, the proportion of college students majoring in art in urban areas is larger than that of students taking other majors. Most students are pampered by their parents since childhood, and their material needs can be basically met. Therefore, the art students have a relatively less desire for material pursuit, which is different from students majoring in science and engineering.

### Measures to improve learning motivation

The results above show students of different genders, regions, and disciplines being analyzed. According to the group characteristics of the students, ideological education is conducted in a personalized and scientific way to promote the better development of learning attitude (Campos-Mesa et al., 2019). For example, students’ learning motivation mainly focuses on material pursuit; as a result, schools, society, and families should guide students to avoid “money worship,” and at the same time give appropriate material rewards to improve students’ self-confidence, increase sense of security in learning, and maintain students’ enthusiasm. Besides, practical activities such as engineering experiments and social practice can also promote change in students’ learning motivation (Kuo et al., 2021; NaderAl-Osaimi and Fawaz, 2022).

## Conclusion

Differences in the learning motivation of college freshmen in Northwest China were investigated in this article. The main conclusions are as follows:

(1) The correlation coefficients of various influencing factors of learning motivation are material pursuit, fear of failure, social orientation, knowledge pursuit and progress, small group orientation, and personal achievement from the top to the bottom.
(2) The learning motivation of the freshmen showed significant differences in gender and whether they are an only child or not, but the differences were not significant in region. Boys’ desire to achieve results in personal achievement is obviously higher than that of girls’, and girls’ pursuit of knowledge is stronger than that of boys.
(3) Significant differences between engineering students and those taking up art-related subjects are reflected in the orientation of seeking knowledge. Students majoring in science and engineering are lower than students majoring in art in seeking knowledge, and students majoring in art have lower desire for material things than students majoring in science and engineering.
(4) Rural students have a higher learning motivation in social orientation, material pursuit, personal achievement, and small group orientation than urban students.

## Data availability statement

The raw data supporting the conclusions of this article will be made available by the authors, without undue reservation.

## Ethics statement

The studies involving human participants were reviewed and approved by the Ethics Committee of Xi’an University of Architecture and Technology. The patients/participants provided their written informed consent to participate in this study.

## Author contributions

KQ drafted the manuscript, designed the questionnaires, and proposed the idea. PG translated and revised the manuscript. RX collected the data, preformed the statistical analysis, and designed the study. BL and XC contributed to the revision of the manuscript and funded the investigation. All authors have read and agreed to the final version of the manuscript.
